# 3D ED/MicroED entering a new era

**DOI:** 10.1107/S205322962400490X

**Published:** 2024-05-31

**Authors:** Mauro Gemmi

**Affiliations:** a Electron Crystallography, Istituto Italiano di Tecnologia, Viale Rinaldo Piaggio 34, Pontedera, Italy

**Keywords:** microcrystal electron diffraction, MicroED, 3D ED, electron diffraction, commentary

## Abstract

Aragon *et al.* [*Acta Cryst.* (2024), C**80**, 179–189], by reporting the discussion and the final conclusions of a round table held during a symposium at the National Center for CryoEM Access and Training, well describe all the advances that have been made for the application of 3D ED/MicroED to pharmaceutical and macromolecular nanocrystals and propose possible future scenarios.

After the development of cryoEM single-particle analysis, the advent of three-dimensional single-crystal electron diffraction (Gemmi *et al.*, 2019 ▸), known as 3D ED/MicroED, was one of the major novelties in crystallography (Service, 2018 ▸). The possibility of a full single-crystal diffraction analysis on nanocrystals has opened a new area of research in crystal structure determination, that since the discovery of X-ray diffraction has remained quite unexplored. Interestingly, the first 3D ED/MicroED experiments date back to almost 20 years ago (Kolb *et al.*, 2007 ▸), but the technique was limited to a few laboratories for almost a decade, until it started to be applied to samples which have an unquestionable value, both commercial and medical, like pharmaceuticals (Jones *et al.*, 2018 ▸; Andrusenko *et al.*, 2019 ▸) and proteins (Nannenga *et al.*, 2014 ▸).

Aragon *et al.* (2024 ▸), by reporting the discussion and final conclusions of a round table held during a symposium at the National Center for CryoEM Access and Training, well describe all the advances that have been made for the application of the technique to these sample types. The biggest issue that is still limiting the diffusion of the method is the request of putting together the know-how of an electron microscopist and a crystallographer for setting up procedures that should work on instruments, the TEMs, that are mainly designed for imaging. This issue is partially mitigated by the availability of data collection software packages that can be applied to simplify any 3D ED/MicroED experiment in most TEMs, and also by the appearance on the market of dedicated electron diffractometers, in which the data collection procedure is partially automated (Simoncic *et al.*, 2023 ▸; Truong *et al.*, 2023 ▸). Both these solutions make 3D ED/MicroED more friendly for crystallographers that are used to X-ray diffraction; however, it is unquestionable that the formation and training of electron crystallographers is of great importance for every laboratory that aims to set up a 3D ED/MicroED facility.

Luckily, as can be seen by the initiative of the National Center for CryoEM, there is a strong tradition among electron crystallographers in organizing teaching events which dates to the nineties of the last century, thanks to the endless effort of Professor Sven Hovmöller. It is therefore common that a lot of schools and workshops are set up every year on different continents. Both the IUCr and ECA strongly support these initiatives: last year an electron crystallography school was organized as a side event of the IUCr Congress in Melbourne, while this year, an electron crystallography school will be held in Padova, Italy, as a side event of ECM34, and finally next year, in Erice, Italy, the International School of Crystallography will be devoted to electron crystallography, as happens every seven years. Finally, thanks to the great effort of Tatiana Gorelik, ELECTRA was founded last year in Europe (https://electraec.wordpress.com/), which is a non-profit educational association with the scope of supporting the organization of schools and workshops related to electron crystallography.

From the experimental point of view, Aragon *et al.* (2024 ▸) correctly identify the full automation of the data collection procedure, following the road opened by cryoEM, as one of the major expected developments. If the fast data collection time of 3D ED/MicroED could be coupled with automation, the method will become appealing for sample screening and quality control. At the same time, the crystallization procedures should be adapted to a new crystal size range which is well below one micron. This will require new crystallization recipes, especially in the case of macromolecules, where the search for ‘large’ crystal size was the only driving force.

Another interesting aspect, still scarcely explored, concerns spatially resolved crystallography, a field where 3D ED/MicroED has a lot to say. In 3D ED/MicroED, the beam size can be reduced to a few nanometres allowing structure variations to be sampled on the same scale (Passuti *et al.*, 2023 ▸). If we consider that 3D ED/MicroED can already investigate very fundamental and elusive aspects like absolute structure (Klar *et al.*, 2023 ▸) and charge–density phenomena (Olech *et al.*, 2024 ▸), we can imagine that, once coupled with the spatial resolution of the technique, we will assist in the appearance of a new science.

The success of 3D ED/MicroED is now producing a flow of new structures determined and refined every year, which requires that the Crystallographic Information File (CIF) stan­dard be made compatible with the peculiarities of ED data. Aragon *et al.* (2024 ▸) pointed out carefully all the critical issues and the IUCr is working on this problem in collaboration with the standardization committee of the NanED (Electron Nanocrystallography) project (https://naned.eu), a project funded by the EU (grant agreement No. 956099) for training the next generation of electron crystallographers. At the moment, a proposed extension of the CIF dictionary is under revision by NanED scientists and by the Commission on Electron Crystallography of the IUCr. The scientific community must also be aware that 3D ED/MicroED data sets are starting to populate open repositories like Zenodo, where two communities (3DED/MicroED data sets; NanED–Electron nanocrystallography project) are actively depositing their experimental data which are freely available for test and for those who want to practice 3D ED/MicroED data analysis.

As can be seen, 3D ED/MicroED has undergone the first stage of development, with both a theoretical and an experimental solid basis, and it is now entering a new era: an era of growth and expansion in which it will support side-by-side single-crystal and powder X-ray diffraction in most crystal structure determination laboratories.
